# Role of miRNAs in Apoptosis Pathways of Immune Cells in Systemic Lupus Erythematosus

**DOI:** 10.1002/iid3.70124

**Published:** 2025-02-06

**Authors:** Amin Azizan, Elham Farhadi, Seyedeh Tahereh Faezi, Ahmadreza Jamshidi, Majid Alikhani, Mahdi Mahmoudi

**Affiliations:** ^1^ Rheumatology Research Center Tehran University of Medical Sciences Tehran Iran; ^2^ Research Center for Chronic Inflammatory Diseases Tehran University of Medical Sciences Tehran Iran

**Keywords:** apoptosis, autoimmunity, microRNAs (miRNAs), systemic lupus erythematosus (SLE)

## Abstract

**Background:**

Systemic lupus erythematosus (SLE) is a chronic autoimmune disease characterized by dysregulated immune responses and multi‐organ involvement. Dysregulation of apoptosis, a key process for maintaining immune homeostasis, plays a critical role in the pathogenesis of SLE. MicroRNAs (miRNAs), small non‐coding RNAs that regulate gene expression, have emerged as important modulators of apoptosis in immune cells, influencing the balance between immune tolerance and autoimmunity.

**Objectives:**

This review aims to comprehensively summarize recent advancements in understanding the roles of miRNAs in apoptosis regulation within immune cells in SLE, highlighting their therapeutic potential for restoring immune balance and mitigating disease progression.

**Results:**

Aberrant expression of specific miRNAs contributes to the dysregulation of apoptosis in SLE immune cells. Pro‐apoptotic miRNAs, such as miR‐125b and miR‐150, are often downregulated, leading to enhanced survival of autoreactive immune cells. Conversely, anti‐apoptotic miRNAs, including miR‐21, are upregulated, further disrupting the delicate balance of immune cell apoptosis. Dual‐function miRNAs, such as miR‐155, exhibit context‐dependent roles based on cellular environments and target gene interactions. This dysregulation promotes the persistence of autoreactive immune cells and the development of autoimmunity.

**Conclusions:**

miRNAs play critical roles in modulating apoptosis pathways, making them promising therapeutic targets for SLE. Restoring the balance of pro‐apoptotic and anti‐apoptotic miRNAs could help reinstate immune tolerance and reduce tissue damage. Future research should focus on elucidating miRNA targetomes, improving delivery systems, and addressing off‐target effects to fully harness their therapeutic potential.

## Graphical abstract

miRNAs play crucial roles in dysregulated apoptosis in SLE immune cells. Most pro‐apoptotic miRNAs are elevated in SLE T cells, contributing to increased apoptosis. Most anti‐apoptotic miRNAs are upregulated in B cells. Promoting survival in SLE, miRNA‐based therapies offer promising strategies to restore apoptotic balance in SLE. Targeted delivery and gene editing enhance miRNA therapeutic potential in SLE.

## 1 Introduction

Systemic lupus erythematosus (SLE) is a chronic autoimmune disease characterized by dysregulated immune responses and systemic involvement, leading to multi‐organ damage and autoantibody production [1, 2]. Although the exact etiology of SLE remains elusive, genetic and environmental factors are known to contribute to its onset [3, 4]. One hallmark feature of SLE is the production of autoantibodies that form immune complexes (ICs), which deposit in tissues and trigger inflammation [5]. Dysregulated apoptosis and impaired clearance of apoptotic cells play a pivotal role in the pathogenesis of SLE by exposing nuclear autoantigens that drive autoantibody production and exacerbate autoimmune responses [6, 7]. Various immune cells in SLE exhibit abnormalities in apoptotic pathways. While B cell resistance to apoptosis encourages the survival of autoreactive clones that generate harmful autoantibodies, enhanced apoptosis in T cells releases autoantigens, causing persistent inflammation. Additionally, defects in apoptotic cell clearance by phagocytes contribute to the accumulation of apoptotic debris and persistent immune activation [8, 9].

SLE is also characterized by defective apoptotic cell clearance, which contributes to the buildup of cellular debris and the exposure of nuclear autoantigens that cause autoimmunity [10]. Macrophages from SLE patients exhibit impaired phagocytosis [11], reduced size, delayed engulfment of apoptotic material [12], and diminished adherence due to lower expression of adhesion receptors like CD44 [13].

CD34^+^ hematopoietic stem cells in SLE also show reduced differentiation into macrophages, exacerbating defective clearance [14].

In germinal centers, tingible body macrophages (TBMs) responsible for apoptotic lymphocyte clearance are reduced in number, leading to the accumulation of nuclear debris [15]. This promotes the activation of autoreactive B cells and T follicular helper cells, resulting in the production of autoantibodies such as anti‐dsDNA, anti‐Sm, and anti‐C1q, which are signs of SLE autoimmunity [16].

These findings are supported by evidence from mouse models, where mutations in genes such as *c‐Mer* or *DNase 1* hinder the removal of apoptotic cells and cause the generation of autoantibodies [17, 18]. Furthermore, the accumulation of apoptotic debris activates Toll‐like receptors (TLRs), specifically TLR2, TLR7, and TLR9, exacerbating autoantibody production and inflammation [19, 20].

These processes collectively highlight how defective apoptotic cell clearance contributes to the breakdown of immune tolerance, sustained inflammation, and tissue damage in SLE.

Recent advances in epigenetics have underscored the role of microRNAs (miRNAs), small non‐coding RNAs that regulate gene expression, as key modulators of apoptotic pathways [21, 22]. By targeting genes involved in apoptosis, miRNAs exert significant control over immune cell survival and death. Certain miRNAs can stimulate or inhibit apoptosis in immune cells, which contributes to the pathophysiology of the illness. Furthermore, dysregulation of miRNAs has been linked to the abnormal apoptotic processes seen in SLE [23, 24, 25].

This review explores the emerging role of miRNAs in regulating apoptosis in the immune cells of SLE patients. We focus on how the dysregulated expression of pro‐apoptotic and anti‐apoptotic miRNAs disrupts immune cell homeostasis, driving the autoimmune responses characteristic of SLE. Furthermore, we discuss the potential therapeutic applications of miRNA modulation to restore proper apoptotic mechanisms and mitigate SLE pathology.

## 2 Apoptosis and Related miRNAs

**Figure 1:**
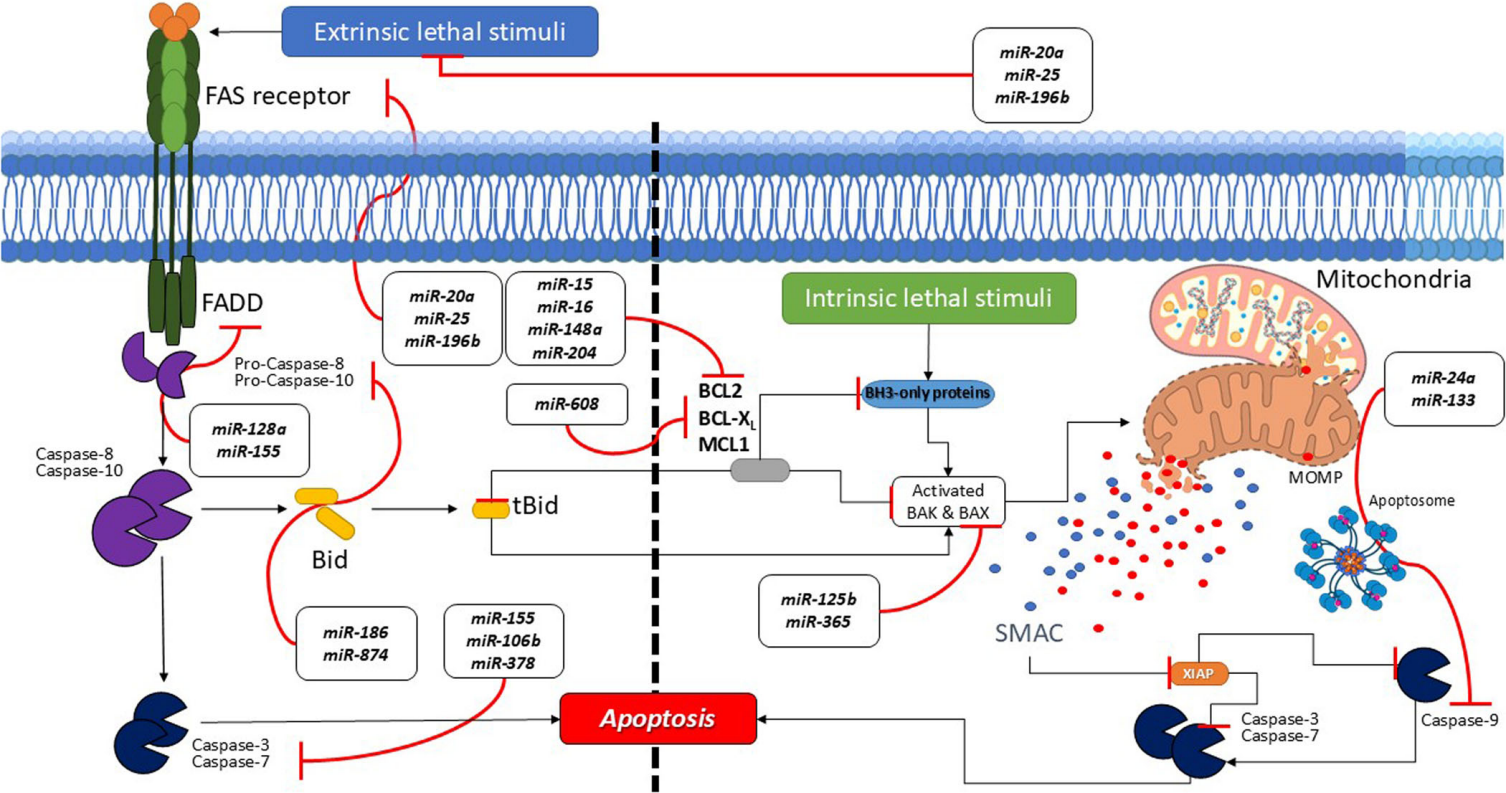
Intrinsic and extrinsic signaling pathways of apoptosis and the effects of miRNAs on these pathways. The two main pathways of apoptosis are extrinsic and intrinsic. Each requires specific triggering signals to begin an energy‐dependent cascade of molecular events. Each pathway activates its initiator caspase [8, 9, 10] which in turn the executioner caspase‐3 will be activated. The exogenous pathway is initiated when a death ligand binds to a death receptor (DR). The release of cytochrome c into the cytoplasm is caused by the activation of the intrinsic or mitochondrial pathway. Apaf1, apoptotic protease activating factor 1; Bak, Bcl‐2 homologous antagonist killer; Bax, Bcl‐2‐like protein 4; Bcl‐2, B‐cell lymphoma 2; Bcl‐xL, B‐cell lymphoma‐extra‐large; Bid, BH3 interacting‐domain death agonist; c‐FLIP, cellular FLICE (FADD‐like IL‐1β‐converting enzyme)‐inhibitory protein; FADD, Fas‐associated death domain protein; FasL, Fas ligand; Smac, second mitochondria‐derived activator of caspases, also referred to as DIABLO; tBid, truncated Bid; TNFR, TNF receptor 1; TRAIL, tumor necrosis factor (TNF)‐related apoptosis‐inducing ligand; XIAP, X‐linked inhibitor of apoptosis.

Apoptosis is a tightly regulated process crucial for maintaining cellular homeostasis and immune system integrity. It eliminates damaged, infected, or excess cells, preventing autoimmune responses and tissue damage. In SLE, apoptosis process is dysregulated and it contributes to the disease's pathogenesis by disrupting immune tolerance, promoting the survival of autoreactive lymphocytes, and increasing the release of autoantigens from dying cells. Apoptosis process predominantly mediated by two major pathways: the intrinsic (mitochondrial) and extrinsic (death receptor) pathways, both of which are dysregulated in SLE [26, 27] (Figure 1).

miR‐708 stimulates apoptosis by regulating the expression of cellular FLICE (FADD‐like IL‐1β‐converting enzyme)‐inhibitory protein (c‐FLIP), which prevents the death‐inducing signaling complex (DISC) formation, and caspase‐8 and caspase‐10 activation [7, 28].

miR‐874, miR‐9, miR‐124, miR‐150, and miR‐125b inhibit cell growth and induces apoptosis by targeting STAT3. STAT3 induces cell survival by increasing the expression of anti‐apoptotic proteins like Bcl‐xL, Bcl‐2, and Mcl‐1 [29, 30].

SOX9 is one of miR‐224 and miR‐134 targets, a DNA‐binding protein that belongs to the high mobility group (HMG) box family of the sex‐determining region Y‐type (SRY). SOX9 inhibits the production of apoptosis‐regulating genes by interacting with the *TNFRSF1b*, *FADD*, *TNFRSF10a*, *TNFRSF10b*, and *RIPK1* promoter regions. Additionally, experimental reduction of SOX9 levels has been demonstrated to restore apoptosis signaling through stimulation of external cell death pathways [31, 32].

Studies have demonstrated that overexpressing miR‐204 leads to an elevation in caspase‐3 expression by inhibiting the expression of ERK1/2 in the MAPK pathway, accompanied by a decrease in both Bcl‐2 levels and the Bcl‐2/Bax ratio [33].

A decrease in XIAP (X‐linked inhibitor of apoptosis) expression by miR‐200c and miR‐142 in inflammatory cytokine‐treated nucleus pulposus (NP) cells and tissues is directly related to excessive apoptosis and an imbalance between extracellular matrix anabolic and catabolic factors [34].

miR‐15 and miR‐16 play a crucial role in post‐transcriptional repression of the *BCL2* gene. Specifically, the data suggest a direct interaction between miRNAs and the 3′‐UTR regions of the *BCL2* gene. This interaction leads to the repression of BCL2 expression [35].

Moreover, miR‐16, miR‐126, miR‐155, miR‐494, miR‐21, miR‐145, and miR‐129c were found to directly target *AKT3* [30, 36], a gene associated with cell development and apoptosis inhibition by inactivating BAD [37] and FOXO [38].

The overexpression of Mcl‐1 partially counteracts the apoptosis and reduced cell viability induced by miR‐137 and miR‐19b, highlighting the regulatory interplay between these miRNAs and Mcl‐1 in cell survival pathways [39].

miR‐186 can directly target and suppress SKP2. Furthermore, these effects were attributed to the inactivation of the PI3K‐Akt pathway and the induction of apoptosis [40].

miR‐181, miR‐137 and miR‐19b effectively target the 3′‐UTRs of three key members within the Bcl‐2 family members: Bcl‐2, Bcl‐2‐L11, and MCL‐1. This regulatory mechanism positions these miRNAs as a pro‐apoptotic agent, modulating cellular responses to apoptotic stimuli [41].

miR‐7 can inhibit apoptosis through the regulation of TNF‐related apoptosis‐inducing ligand (TRAIL) [42]. TRAIL induces apoptosis by binding to DRs [43].

miR‐128a regulates Fas‐associated via death domain (FADD) and can also target caspase‐3. Expression of miR‐128a causes apoptosis resistance by targeting FADD [44].

miR‐365 directly targets Bcl‐2 associated X, apoptosis regulator (Bax) [45] and Src homology 2 domain‐containing 1 (SHC1) adaptor protein [46]. It has been reported that SHC1 interacts with Grb2‐associated binding protein 1 (GAB1), a signaling pathway known to activate PI3K/Akt to inhibit the apoptosis process [47]. Moreover, miR‐365, miR‐125a, miR‐654, miR‐140, miR‐145, miR‐21, and miR‐26 can target Bax and cause apoptosis inhibition [30]. In addition, miR‐380, miR‐30a, miR‐125b, and miR126 target p53 and inhibit its apoptosis‐inducing function [48, 49].

Certain miRNAs exhibit dual functions, capable of either promoting or inhibiting apoptosis, contingent upon the cellular milieu or the presence of particular targets.

Through gain‐ and loss‐of‐function experiments, miR‐155 inhibition reduces while its overexpression causes IL‐1β‐induced apoptosis. PIK3R1, a distinct regulatory component of the PI3K/Akt pro‐survival pathway, is a direct target of miR‐155. Restoring PIK3R1 expression reversed the pro‐apoptotic effects of miR‐155, similar to treatment with an inhibitor of Akt. Therefore, miR‐155 appears to promote cell death by interfering with PI3K/Akt signaling through direct suppression of the pathway's PIK3R1 component [50]. On the other hand, in psoriasis, miR‐155 can stimulate cell proliferation and suppress apoptosis through targeting phosphatase and tensin homolog (PTEN) [51].

Various contexts have identified miR‐150 as a critical regulator of apoptosis. The cardioprotective effects of miR‐150 during ischemic injury are partially mediated by its direct repression of pro‐apoptotic genes such as *egr2*, which induces apoptosis by direct transactivation of BNIP3L and BAK [52], and p2x7r, which induces apoptosis by activating caspases 3 and 7 [53, 54]. Conversely, overexpression of miR‐150 has been shown to enhance apoptosis by regulating c‐Myb [55].

As a result of miR‐126 expression, the transcriptional activity of p53 can be reduced and apoptosis inhibited through the downregulation of multiple p53‐related target genes [56, 57]. On the other hand, miR‐126 is capable of inducing apoptosis, indicating its involvement in regulating cell death processes. Its targeting of downstream effectors within the PI3K/PDK1/AKT pathway evidences this. Also, miR‐126 upregulates Bad and Bax proteins. This upregulation leads to increased caspase 3/7 activity, ultimately promoting apoptosis [58].

Suppression of miR‐494 unlocks inhibition of the pathway, enabling heightened PI3K, AKT, and mTOR phosphorylation. By binding target transcripts and suppressing the translation of regulators in this signaling network, miR‐494 exhibits the capacity to adjust apoptosis activation through control of PI3K/AKT/mTOR activity governing cell survival versus death pathways [59]. On the other hand, studies have demonstrated that miR‐494 engages with the ASK‐1/STRAP/14‐3‐3 complex, thereby promoting the activation of TNF‐/ASK‐1‐mediated apoptosis [60]. miR‐125b can lower the expression of the *STAT3* gene, which can raise the expression of the Bcl‐2 family and work as a pro‐apoptotic miRNA [61]. Also, it has been shown that miR‐125b can target P53 and inhibit the apoptosis process in lens epithelial cells [62].

miR‐21 suppresses PTEN, leading to the stimulation of PI3K/AKT [63]. PTEN represses PI3K/AKT to suppress cell proliferation. As a result of inhibiting PIP3 kinase phosphorylation, PTEN induces apoptosis and blocks Akt activity [64]. Therefore, miR‐21 can act as an anti‐apoptotic miRNA by inhibiting PTEN. On the other hand, studies have shown that miR‐21 can target Bcl‐2 in some cases and act as a pro‐apoptotic agent [65, 66, 67].

**Figure 2:**
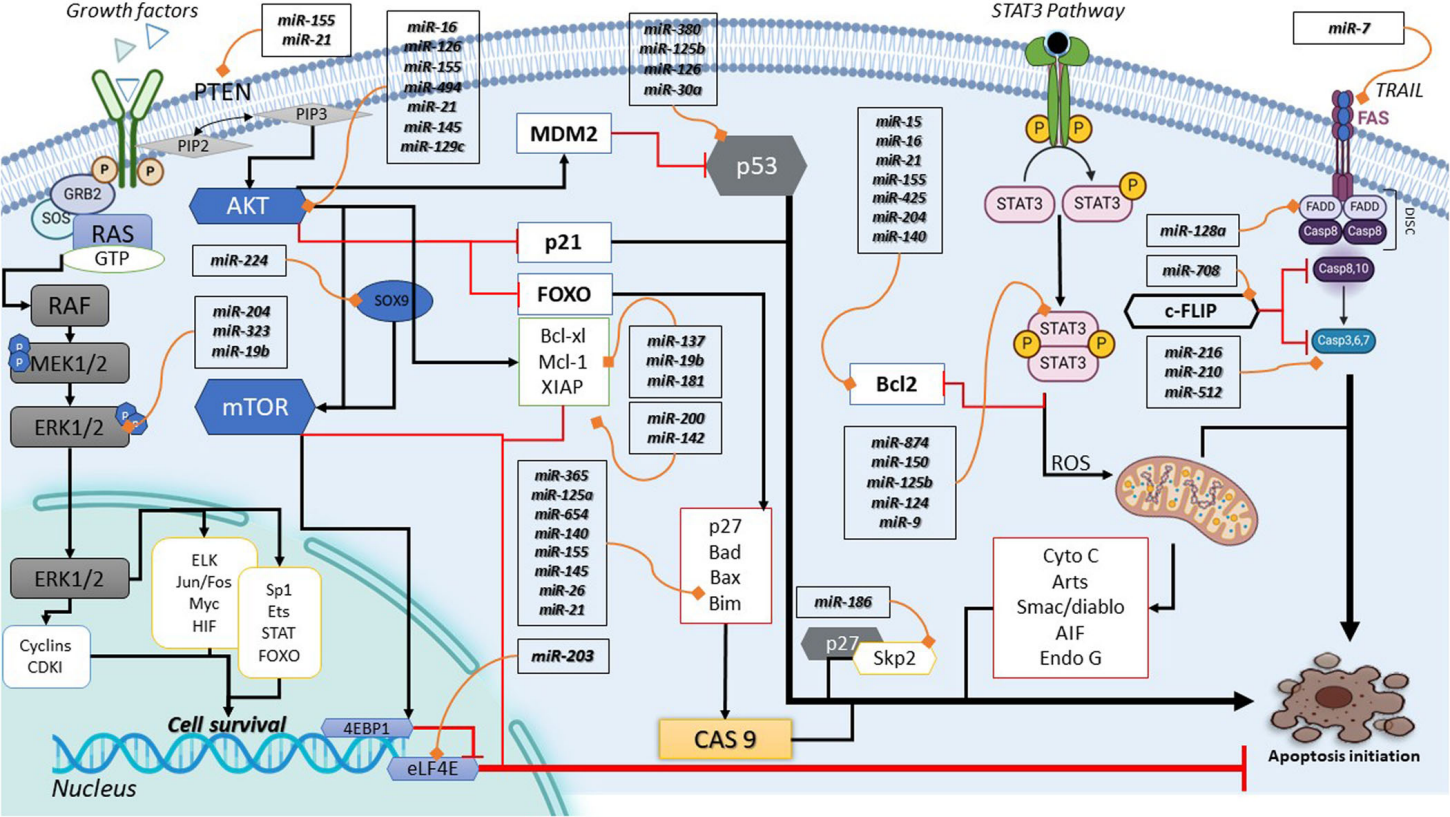
Schematic overview of miRNA regulation in apoptosis via key signaling pathways. This figure provides a comprehensive view of the intricate role of microRNAs (miRNAs) in modulating apoptosis through various signaling pathways. It showcases miRNAs as pivotal regulators that either promote or inhibit apoptosis by interacting with the PI3K/AKT, JAK/STAT3, TRK, and TRAIL/FADD pathways. The diagram illustrates how specific miRNAs target components within these pathways, such as upregulating or downregulating the PI3K/AKT pathway which is crucial for cell survival and proliferation. Similarly, it depicts the modulation of the JAK/STAT3 pathway by miRNAs, affecting inflammatory responses and cell fate. The TRK pathway, known for its role in neuronal survival, is also influenced by miRNA activity, as is the TRAIL/FADD pathway which is central to the extrinsic apoptotic process. AKT, protein kinase B; BCL‐2, B‐cell lymphoma 2; BCL‐XL, B‐cell lymphoma‐extra‐large; C‐FLIP, cellular FLICE‐inhibitory protein; ERK, extracellular signal‐regulated kinase; FAS, fatty acid synthase; FOS, FBJ murine osteosarcoma viral oncogene homolog; FOXO, forkhead box O; GRB2, growth factor receptor‐bound protein 2; GTP, guanosine triphosphate; JUN, Jun proto‐oncogene; MAPK, mitogen‐activated protein kinase; MCL‐1, myeloid cell leukemia 1; MDM2, mouse double minute 2 homolog; MEK, mitogen‐activated protein kinase kinase; mTOR, mechanistic target of rapamycin; PIP, phosphatidylinositol phosphate; PTEN, phosphatase and tensin homolog; RAF, rapidly accelerated fibrosarcoma; RAS, rat sarcoma; RTK, receptor tyrosine kinase; SKP2, S‐phase kinase‐associated protein 2; SOS, son of sevenless; SOX9, SRY‐Box transcription factor 9; STAT3, signal transducer and activator of transcription 3; TRAILL, TNF‐related apoptosis‐inducing ligand; XIAP, X‐linked inhibitor of apoptosis protein.

All miRNAs that may play a role in inducing or preventing apoptosis are shown in Figure 2.

### 2.1 Immune Cells Apoptosis in SLE

The dysregulation of apoptosis in immune cells is a distinctive feature of SLE, significantly contributing to the intricate pathogenesis of this autoimmune disorder [68].

B cells in SLE patients often show increased survival, which may be linked to dysregulated apoptosis [69]. Specifically, upregulation of Bcl‐2, Bcl‐xL, and Mcl‐1 in B cells from SLE patients has been documented, which contributes to their resistance to programmed cell death [70]. Meanwhile T cells generally exhibit a reduction in numbers due to increased apoptosis [71, 72]. A study investigating the apoptosis of T cells in SLE patients exhibited significantly increased apoptosis compared to healthy controls. Specifically, CD4^+^ T cells showed a marked increase in apoptosis, contributing to the decreased CD4/CD8 ratio observed in SLE. The study further revealed that high levels of IL‐10 in SLE serum induced apoptosis through the Fas‐FasL pathway, particularly in CD4^+^ T cells, and blocking IL‐10 reduced apoptosis in these cells [73].

In SLE, neutrophils have an active phenotype characterized by enhanced aggregation and the formation of platelet‐neutrophil complexes, in contrast to neutrophils from healthy controls. Moreover, SLE neutrophils have an increased susceptibility to apoptosis, which contributes to illness development [74].

An investigation measured apoptotic neutrophils in the peripheral blood of SLE patients and discovered that the proportion of apoptotic neutrophils was markedly elevated in SLE patients compared to healthy controls and disease controls, including those with rheumatoid arthritis and inflammatory bowel disease. Neutrophil apoptosis in SLE had a positive correlation with disease activity, as indicated by the SLAM score, and was more severe in individuals with heightened anti‐dsDNA antibody levels. Higher numbers of apoptotic neutrophils and higher Fas expression in the neutrophils were observed in neutropenic patients with SLE [75].

DCs appear to be involved in altered apoptosis regulation [76]. There is evidence suggesting that while some types of DCs (particularly plasmacytoid DCs) can exhibit defects in clearing apoptotic cells, myeloid DCs tend to show an impaired apoptotic response. This dysfunction may contribute to prolonged survival and increased activation of DCs, which exacerbates the inflammatory environment in SLE. Specifically, defective clearance of apoptotic material by DCs can lead to an accumulation of self‐DNA and RNA, further driving type I interferon production and autoimmunity [77, 78].

Further, in SLE, monocytes and macrophages play crucial roles in immune dysregulation, with their apoptosis and clearance functions being altered in ways that exacerbate disease pathogenesis [79, 80]. While there is no consensus suggesting that these cells undergo “more” or “less” apoptosis across all contexts, the primary issue appears to be a disruption in their ability to effectively clear dying cells, contributing to a build‐up of autoantigens that drive autoimmune responses.

However, it is important to note that the mechanisms behind these changes are complex and not solely attributable to apoptosis dysregulation [81]. While altered apoptosis likely contributes to immune cell dysfunction in SLE, it should not be oversimplified as the primary cause.

### 2.2 Pro‐Apoptotic miRNAs in the Immune Cells of SLE

As mentioned, miR‐150 can induce apoptosis by targeting STAT3 [29]. Additionally, splenic DCs from SLE mice showed significantly lower levels of miR‐150, which targets the triggering receptor produced by myeloid cells (TREM‐1). TREM‐1 is known to foster inflammation and impede apoptosis via the PI3K/Akt pathway, which causes caspase 3 regulation, potentially perpetuating a chronic inflammatory cellular state [82]. TREM‐1 is a key amplifier of inflammatory responses, particularly during bacterial or fungal infections. Its overexpression has been linked to increased apoptosis. Mechanistically, TREM‐1 overexpression downregulates the anti‐apoptotic gene *Bcl‐2* while upregulating pro‐apoptotic genes such as *Bax*, and cleaved caspase‐3, and cleaved caspase‐9 proteins [83]. Through this cascade, DCs may evade apoptosis, thereby potentially maintaining their survival and enhancing antigen presentation, which could cause further inflammation. This prolonged survival and activity of DCs might contribute to the persistence of autoreactive T cells, thereby exacerbating systemic autoimmunity and increasing disease severity [76]. Conversely, numerous reports indicate that c‐myb is the target protein of miR‐150. Additionally, a particular circular RNA (circLOC101928570) enhanced PBMC apoptosis by acting as a miR‐150‐5p sponge to alleviate the restrictive impact on its target c‐myb, which regulates the activation of immune inflammatory responses [84]. c‐myb exerts anti‐apoptotic effects by upregulating Bcl‐2 and Bcl‐xL, which prevent mitochondrial‐mediated apoptosis, therefore preserving cell viability, particularly during progenitor cell differentiation [85]. Furthermore, c‐myb can inhibit the production of pro‐apoptotic genes like *Bim*, hence enhancing cell survival [86]. Another significant facet of c‐myb's function in apoptosis is its connection with the p53 pathway. c‐myb regulates the production of p53, a crucial modulator of apoptosis, and may either enhance or suppress p53‐mediated apoptotic responses based on the cellular context and associated signaling pathways [87]. miR‐150 exhibits high expression levels in mature T lymphocytes in human SLE patients, while the c‐myb/IL2R axis serves as a crucial transcription factor involved in regulating lymphocyte development and contributing to the pathogenesis of SLE. Individuals with SLE significantly reduced the expression of IL2RA on Th1, Th2, Tc1, and Tc2 T cell subsets compared to healthy controls [84]. Also, studies show that c‐myb is important for the growth of T cells and B cells [88, 89, 90, 91, 92], and interaction between miR‐150 and c‐myb is important for embryonic development [93]. Dysregulation in T or B cell development may result in the breakdown of immunological tolerance, facilitating the persistence of autoreactive cells. This malfunction leads to the characteristics of SLE, such as elevated autoantibody synthesis, irregular cytokine release, and persistent systemic inflammation [94].

Besides in immune cells, studies on miR‐150 levels in SLE have shown conflicting results. For instance, some research indicates that miR‐150 levels in plasma are reduced in SLE patients compared to healthy controls, suggesting its potential as a biomarker for disease activity [95]. However, other studies report elevated levels of miR‐150 in plasma, particularly in active SLE cases, indicating a complex role in the disease's pathogenesis [96].

The significantly increased expression of miR‐410 in CD4^+^ T cells of SLE patients compared to healthy controls suggests a potential association between miRNA‐410 levels and the underlying mechanisms of SLE pathogenesis. MiR‐410 regulates by inhibiting the transcriptional activity of STAT3 by direct binding to the 3′‐UTR of STAT3 mRNA [97]. Furthermore, inhibition of STAT3 resulted in a reduction in IL‐10 expression in T cells. In SLE, IL‐10 production and secretion are dysregulated, which can disrupt T cell homeostasis and increase B cell survival and autoantibody formation, resulting in an imbalance in immunological regulation [98, 99]. In addition, STAT3, recognized for its role in promoting cell survival, controls the production of Bcl‐xL, Bcl‐2, and Mcl‐1. Furthermore, the multifaceted role of STAT3 extends to modulating key apoptotic pathways, notably the PI3K/Akt pathway. This intricate regulatory interplay between miR‐410, STAT3, and the apoptotic pathways elucidates a potential mechanistic link underlying the aberrant apoptotic processes observed in SLE, highlighting the significance of the miR‐410‐STAT3 axis in the context of SLE pathogenesis [100]. However, miR‐410 expression is significantly decreased in PBMC samples from SLE patients relative to healthy controls, and miR‐410 was demonstrated to control 17 of the 36 anticipated target genes, highlighting its essential role in key pathways, including those associated with cancer, that contribute to the pathogenesis of SLE [101]. Notably, whereas miR‐410 levels are decreased in PBMCs [101], studies on T cells from SLE patients have indicated elevated expression of this miRNA [100]. This disparity indicates that different cell types within PBMC populations, such as monocytes or B cells, may affect total miR‐410 levels. Nonetheless, the precise roles of these cells have still to be investigated, underscoring the necessity for more research into cell‐type‐specific miRNA regulation in SLE.

The lower expression of miR‐125b detected in SLE patients’ PBMCs corresponds with the existence and development of the illness. Furthermore, exposure to ultraviolet B (UVB) radiation diminishes miR‐125b levels in PBMCs, indicating that UVB aggravates SLE through the modulation of miRNA expression. The UV radiation resistance‐associated gene (UVRAG), an essential regulator of autophagy, was recognized as a direct target of miR‐125b [102]. In addition, as mentioned before, miR‐125b exerts regulatory control by directly targeting key transcription factors such as STAT3 and ETS1 via the AK1/STAT3 pathway, which can cause apoptosis [103]. ETS1 can cause apoptosis resistance by inducing BCL‐xL expression [104] and polymorphism in *ETS1* gene may be related to SLE [105]. Moreover, the expression patterns of various miRNAs in neutrophils and monocytes indicate cell‐type‐specific dysregulation in SLE. Significantly, although the expression of the majority of miRNAs in neutrophils was unchanged relative to healthy donors, miR‐125b exhibited a substantial increase. Conversely, monocytes from individuals with SLE demonstrated reduced expression of miR‐125a [106, 107]. Such cell‐specific dysregulation of miRNAs may contribute to distinct functional impairments in these immune cells, further driving the chronic inflammatory and autoimmune processes characteristic of SLE.

Significantly decreased levels of miR‐15b were observed in B cells from both SLE patients and lupus‐like mice. Additionally, it has been established that cyclin D3 (CCND3), a critical player in the inhibition of apoptosis, is directly targeted by miR‐15b, and induced CCND3 expression may contribute to the abnormality of B cells in SLE [108]. This interaction is significant considering the role of the CCND3‐CDK6 complex's kinase activity, which, while conferring an anti‐apoptotic function, can also instigate the production of reactive oxygen species (ROS), potentially initiating the apoptotic cascade [109]. Therefore, the decreased expression of miR‐15b in SLE B cells may lead to the upregulation of CCND3, functioning as an anti‐apoptotic protein and contributing to apoptosis resistance in these cells.

Furthermore, circulating miR‐15b (as a B cell‐related miRNA) in plasma has demonstrated higher levels in comparison to controls [95].

The reduced expression of miR‐99a‐3p was observed in B cells of individuals diagnosed with SLE compared to healthy controls. Upon transfection with miR‐99a‐3p, Ball‐1, Jurkat, and THP‐1 cells displayed a notably increased apoptosis rate by targeting EIF4EBP1, a negative regulator of mRNA translation and mTOR [110]. EIF4EBP1, as a target gene of the oncoproteins ETS1 and MYBL2, may influence apoptosis by promoting the survival of tumor cells in unfavorable situations. ETS1 and MYBL2 are associated with angiogenesis regulation, with ETS1 regulating VEGF production, crucial for neovascularization and tumor proliferation. The overexpression of MYBL2, sustained by hypoxia‐inducible factor 2α (HIF‐2α), enhances cell viability under hypoxic circumstances by safeguarding cells against hypoxia‐induced apoptosis [111]. Therefore, the dysregulation of miR‐99a‐3p in SLE B cells may contribute to altered apoptosis regulation, as the downregulation of miR‐99a‐3p leads to increased EIF4EBP1 expression, potentially enhancing cell survival and resistance to apoptosis in unfavorable conditions.

It is important to note that while most pro‐apoptotic miRNAs increased in their levels in SLE T cells or PBMCs, this elevation in pro‐apoptotic miRNAs contributes to the increased apoptosis rate, further influencing SLE pathogenesis. However, studies demonstrate pro‐apoptotic miRNA rates decrease in B cells as these cells have more survival in SLE conditions. The multifaceted role of miRNAs demonstrates their potential as therapeutic targets for modulating immune responses and apoptosis pathways in SLE.

### 2.3 Anti‐Apoptotic miRNAs in Immune Cells of SLE

miR‐181 in SLE impacts MAPK8 signaling. Reduced miR‐181 expression was observed in SLE patients’ PBMCs and MRL/lpr mice. The increased expression of miR‐181 demonstrated anti‐apoptotic effects in vitro and mitigated kidney injury in vivo. These effects were attributed to the targeting of MAPK8 by miR‐181, leading to the inhibition of phosphorylation of p38 and p44/42 [112].

miR‐137 is downregulated while AMP‐activated protein kinase (AMPK) was upregulated in SLE CD4^+^ T cells compared to controls. Notably, miR‐137 targeted AMPK directly. Overexpression of miR‐137 suppresses apoptosis and NLRP3 inflammasome activity in primary CD4^+^ T cells. In contrast, miR‐137 inhibition causes an apoptosis increase [113]. AMPK mediates its pro‐apoptotic effects primarily by modulating various downstream signaling events, including the regulation of c‐Jun N‐terminal protein kinase (JNK) and p53, inhibiting mTORC1, or directly enhancing the activation of pro‐apoptotic proteins, among other pathways [114, 115]. However, as mentioned, miR‐181 and miR‐137 effectively target three Bcl‐2, Bcl‐2‐L11, and MCL‐1 and can act as apoptotic miRNAs [41].

Reduced levels of miR‐145 have been observed in SLE patients' T cells compared to healthy controls. Functional studies demonstrated that overexpression of miR‐145 in Jurkat cells led to a significant reduction in STAT1 levels, identifying STAT1 as a direct target of miR‐145 [116]. STAT1 has a dual role in apoptosis, acting as both an inducer and an inhibitor depending on the context. As a pro‐apoptotic factor, STAT1 mediates interferon responses by promoting the expression of Fas/FasL, TRAIL, and mitochondrial regulators like PUMA and BAX, leading to caspase activation and cell death. It also enhances DNA damage responses via p53‐dependent pathways [117, 118]. Conversely, STAT1 can suppress apoptosis by upregulating survival genes such as *Bcl‐2* and *Bcl‐xl* and promoting anti‐apoptotic cytokines like IL‐10. In certain contexts, such as tumor resistance or immune regulation, STAT1 activates the PI3K/Akt pathway, supporting cell survival [119]. Furthermore, as mentioned before, miR‐145 can target Bax and cause apoptosis inhibition [30] and target AKT3, causing apoptosis induction [30, 36]. Thus, the dysregulation of miR‐145 may disrupt the delicate equilibrium between T cell survival and apoptosis in SLE, potentially contributing to the persistence of autoreactive lymphocytes or the excessive loss of regulatory T cells, both of which are hallmarks of SLE pathogenesis [120].

The expression of miR‐152‐3p doubled in B cells of SLE patients compared to healthy controls. Analysis revealed that KLF5 is a target of miR‐152‐3p, whose elevated levels in SLE B cells result in a reduction of KLF5 expression. Moreover, KLF5 was found to bind to the BAFF promoter and regulate its expression in B cells. Consequently, BAFF expression significantly decreased in SLE B cells upon KLF5 overexpression compared to the control group [121]. Moreover, reduced BAFF signaling attenuates the noncanonical NF‐kB pathway, hindering B cell proliferation despite the simultaneous activation of a protective p53 response [122].

Decreased levels of miR‐633 were evident in CD4^+^ T cells from individuals with SLE compared to healthy CD4^+^ T cells. Notably, the investigation revealed AKT1 as a direct target of miR‐633 [123]. Furthermore, it has been established that AKT1 has a regulatory effect on mTOR transcription. miR‐633 can inhibit AKT1 and mTOR expression. This inhibition is crucial as mTOR is implicated in the promotion of apoptosis via the activation of the inositol‐requiring protein‐1/c‐Jun N‐terminal kinase (IRE/JNK) pathway. Activation of this pathway triggers caspase‐3 and suppresses the expression of Bcl‐2, ultimately inducing apoptosis [124].

In many studies, miR‐21 has been predominantly described as an anti‐apoptotic miRNA in autoimmune conditions [67]. Elevated levels of miR‐21 were notably observed in T cells of individuals diagnosed with SLE compared to healthy donors [125, 126]. miR‐21 can demonstrate anti‐apoptotic properties via targeting programmed cell death protein 4 (PDCD4), thereby inhibiting apoptosis via modulation of the PI3K/Akt/FOXO1 signaling pathway [127]. In addition, the loss of PDCD4 has been linked to the activation of apoptosis through the enhancement of procaspase‐3 mRNA translation, underscoring the intricate and context‐dependent function of PDCD4 in modulating cell survival and death [128]. Furthermore, investigation indicates that miR‐21 levels are markedly increased in the plasma of SLE patients relative to healthy controls, implying its potential as a biomarker for disease diagnosis and monitoring [129, 130, 131]. These findings collectively underscore the likely involvement of miR‐21 in the pathophysiology of SLE through the modulation of apoptotic pathways.

**Figure 3:**
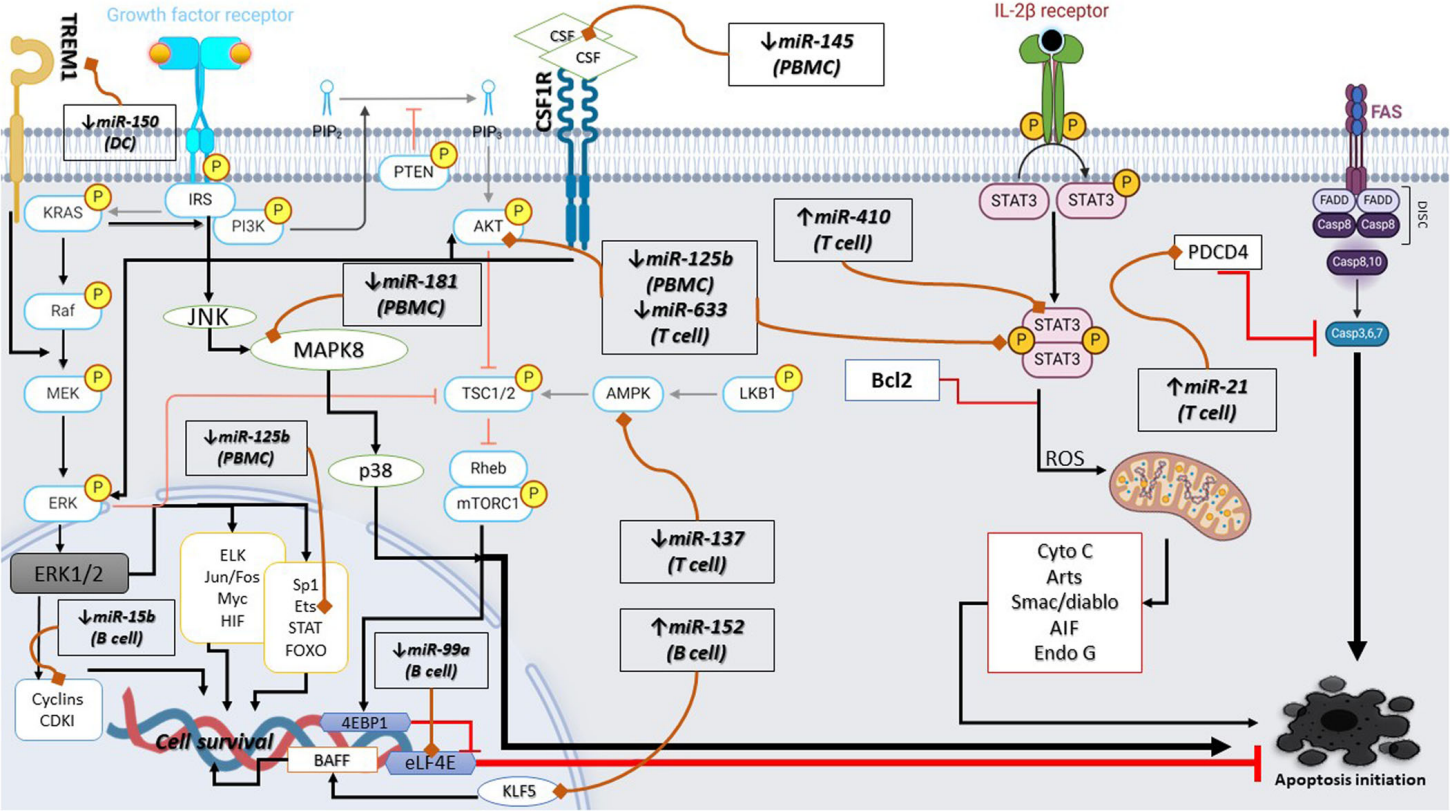
The role of miRNAs in SLE pathogenesis through apoptosis modulation. This figure depicts the complex interplay between microRNAs (miRNAs) and key signaling pathways in the pathogenesis of systemic lupus erythematosus (SLE) by modulating apoptosis. It highlights how miRNAs can influence apoptosis through various pathways. Each pathway is represented by its interaction with specific miRNAs, demonstrating the potential for miRNAs to serve as biomarkers and therapeutic targets in SLE. BAFF, B‐cell activating factor; c‐myb, cellular myeloblastosis protein; JNK, c‐Jun N‐terminal Kinase; KLF5, Krüppel‐like factor 5; PDCD4, programmed cell death protein 4.

Most notably, the majority of these anti‐apoptotic miRNAs are downregulated in SLE T cells and PBMCs, while anti‐apoptotic miRNAs are upregulated in SLE B cells, providing a plausible explanation for the observed high apoptosis rates in SLE T cells and increased survival in B cells, which contributes to the intricate pathogenesis of the disease. miRNAs that play a crucial role in SLE pathogenesis through apoptosis pathways in immune cells are listed in Table 1 (Figure 3).

**Table 1:** Differentially expressed miRNAs play an important role in the apoptosis of immune cells in SLE.

miRNA	Population involved	Sample type	Assessed cell line	Target regulator	mechanism	miRNA status in SLE	Study
*MiR‐181d‐5p*	Human (10 SLE and 10 healthy controls (HCs)), MRL/lpr mice	PBMC	THP‐1	MAPK8	miR‐181d‐5p inhibits MAPK8 and phosphorylation of p38 and p44/42 which cause inhibiting the apoptosis.	Downregulated	[112]
*miR‐137*	Human (20 SLE and 20 HCs)	CD4^+^ T cells	—	AMPK	inhibits CD4 + T cell apoptosis in SLE patients by modulating the AMPK pathway and NLRP3.	Downregulated	[113]
*miR‐145*	Human (26 SLE and 27 HCs)	CD4^+^ T cells	Jurkat cells	STAT1	Targeting STAT1 and acting as both an inducer and inhibitor of apoptosis depending on the context.	Downregulated	[116]
*miR‐99a‐3p*	Human (10 SLE and 10 HCs), mouse MRL/lpr	B cells	Ball‐1, Jurkat, THP‐1 and K562	EIF4EBP1	miRNA‐99a‐3p targets EIF4EBP1 which regulates the activity of mTORC1, thereby induce apoptosis.	Downregulated	[110]
*miR‐152‐3p*	Human (30 SLE and 30 HCs)	B cells	—	KLF5	The KLF5 protein, when bound to the BAFF promoter, exerts regulatory control over its expression in B cells.	Upregulated	[121]
miR‐633	Human (20 SLE and 20 HCs)	CD4^+^ T cells	Jurkat	AKT1	downstream impact of miR‐633 was observed in the inhibition of AKT1 and mTOR expression. This inhibition is crucial as mTOR is implicated in the promotion of apoptosis.	Downregulated	[123]
miR‐21	Human (45 SLE and 30 HCs)	CD4^+^ T cells	—	PDCD4	miR‐21 targets PDCD4 which has been linked to apoptosis induction by promoting the translation of procaspase‐3 mRNA	Upregulated	[125]
miR‐150	eMRLlpr/lpr mice and C57BL/6 wild type mice	Splenic cDCs	—	TREM‐1	miR‐150 regulates TREM‐1 which promotes inflammation and inhibits apoptosis through the PI3K/Akt pathway.	Downregulated	[82]
	Human (62 SLE and 62 HCs)	PBMC, CD4^+^ and CD8^+^ T cells	—	c‐myb	miR‐150 can target c‐myb and induce apoptosis by miR‐150‐5p/c‐myb/IL2RA pathway	Upregulated	[84]
miR‐15	Human (5 SLE and 5 HCs) and C57BL/6 and B6.MRL‐Faslpr/J lupus‐prone mice	B cells	—	CCND3	miR‐15 inhibits CCND3 which acts as an anti‐apoptotic agent by CCND3‐CDK6 complex.	Downregulated	[108]
miR‐410	Human (20 SLE and 20 HCs)	CD4^+^ T cells	—	STAT3	miR‐410 targets STAT3 which relating to anti‐apoptotic agents and PI3K/Akt pathway	Downregulated	[97]
miR‐125b	Human (50 SLE and 26 HCs)	PBMC	—	STAT3, ETS1	miR‐125b targets STAT3 which relating to BCL‐2 family agents and PI3K/Akt pathway and cause apoptosis initiation.	Downregulated	[103]

### 2.4 Therapeutic Potential of Targeting miRNAs

The interplay between defective apoptosis and SLE pathogenesis underscores the importance of targeted therapeutic strategies aimed at restoring proper apoptotic mechanisms to mitigate autoimmune responses in this disorder [132]. While understanding the dysregulation of miRNAs in SLE is critical, their true therapeutic potential lies in the ability to modulate these molecules effectively [133]. Among their diverse roles, miRNAs involved in apoptosis are key targets for restoring immune homeostasis and mitigating autoimmunity.

Lower expression levels of miR‐125b in PBMCs of SLE patients have been linked to impaired regulation of apoptosis through its interaction with STAT3 [103]. Histone deacetylase inhibitors such as valproic acid and butyrate have been shown to upregulate miR‐125b along with other miRNAs, modulating pathways critical for B cell differentiation by suppressing AICDA and PRDM1 expression [134]. These findings highlight the potential for epigenetic interventions in targeting autoimmune responses. However, histone deacetylase inhibitors act on multiple targets, and their phenotype‐modulating effects cannot be attributed solely to miRNA regulation [135, 136].

Extensive studies have highlighted miR‐21 as a critical regulator of apoptosis and immune dysregulation in SLE, making it an attractive therapeutic target. Silencing miR‐21 in vivo using locked nucleic acids (LNAs) effectively reversed splenomegaly, a hallmark of lupus‐like autoimmunity in B6.Sle123 mice. This intervention also restored PDCD4 expression, demonstrating miR‐21's role in modulating apoptotic pathways. Additionally, treatment with anti‐miR‐21 significantly altered the CD4/CD8 T cell ratio, reduced Fas receptor‐expressing lymphocytes, and improved T cell survival, highlighting its potential for broader immune modulation [137].

Further evidence of miR‐21's therapeutic relevance comes from its deficiency in the cGVHD model, where miR‐21‐deficient mice displayed a significant reduction in lupus‐like autoimmunity. These mice exhibited decreased splenomegaly, lower autoantibody titers, and alterations in co‐stimulatory pathways essential for B and T cell activation. Notably, miR‐21 deficiency reduced pro‐inflammatory CD4(+) IL‐17(+) T cells while expanding CD4(+) CD25(+) FoxP3(+) regulatory T cells, indicating a role in rebalancing pro‐ and anti‐inflammatory T cell subsets [138].

In clinical contexts, extracellular vesicles (EVs) derived from the plasma of SLE patients have been shown to carry elevated levels of miR‐21, miR‐29a, and miR‐29b, which can activate TLR7 and TLR8, promoting inflammation. A recent study evaluated a novel therapeutic approach targeting these EV‐encapsulated miRNAs using LNA antagonists. In this study, PBMCs from active SLE patients were pre‐treated ex vivo with a cocktail of LNA inhibitors targeting miR‐21, miR‐29a, and miR‐29b. These modified PBMCs were then transferred into immunodeficient NGS mice to generate humanized mouse models. The inhibition of these miRNAs significantly reduced pro‐inflammatory cytokines, including IFN‐γ, TNF‐α, and IL‐6, and decreased histopathological damage in key target organs such as the small intestine, liver, and kidney. Histological analysis demonstrated reduced immune cell infiltration in these organs, corroborated by a significant reduction in human CD3^+^ T cell infiltrates. These findings suggest that combinational miRNA antagonism using LNA cocktails can suppress autoimmune‐mediated inflammation and alleviate disease progression in SLE [139].

The use of antagomir‐21 has also shown promise in preclinical models. In MRL/lpr mice, this inhibitor reduced the expansion of T follicular helper (Tfh) cells, alleviating disease severity and autoantibody‐mediated immune responses [140]. These findings position Antagomir‐21 as a specific and effective tool for targeting miR‐21 in lupus.

Lastly, miR‐21 is implicated in lupus nephritis. In MRL/lpr mice, the Qihuang Jianpi Zishen decoction (QJZ) targeted the GAS5/miR‐21/Sprouty1 axis and the ERK/CREB signaling pathway, which are critical for glomerular mesangial cell proliferation [141]. Although QJZ alleviated renal damage by modulating miR‐21 pathways, its lack of identified active ingredients and unclear mechanisms of action limits its reliability as a therapeutic option.

The discovery of miRNAs has revolutionized our understanding of gene regulation, with most miRNA‐targeted therapeutic research focused on cancer [142]. However, these therapies also hold significant potential in autoimmune diseases such as SLE, where miRNAs play critical roles in immune dysregulation and apoptosis [143, 144]. While miRNA mimics and anti‐miRNAs offer therapeutic promise, challenges such as off‐target effects, immune toxicity, and inefficient delivery systems have hindered clinical progress [142].

Emerging delivery technologies, including ligand‐conjugated nanoparticles, viral‐based systems, and non‐viral carriers, are advancing the field by enhancing specificity and reducing toxicity. Additionally, novel therapeutic strategies under investigation, such as anti‐miRNA oligonucleotides, miRNA sponges, small molecule inhibitors, and miRNA masking, may expand the applicability of miRNA therapies [145]. These approaches aim to either suppress dysregulated miRNAs or restore the function of suppressed ones, offering a versatile toolbox for addressing complex diseases like SLE.

Computational tools and high‐throughput sequencing have improved miRNA target identification, but functional validation remains a bottleneck due to the complexity of miRNA interactions. Understanding miRNA targets more comprehensively is essential to mitigate risks and improve therapeutic specificity [146].

Currently, miRNAs show the most promise as diagnostic tools, given their stability in circulation and potential for disease monitoring. As research progresses, miRNA‐targeted therapies could become integral to treating autoimmune conditions, complementing siRNA‐ and mRNA‐based approaches and paving the way for precision medicine in SLE.

## 3 Conclusion

The intricate interplay between miRNAs and apoptotic pathways in immune cells plays a crucial role in the pathogenesis of SLE. Dysregulation of miRNAs contributes to the imbalance of apoptosis in various immune cell types, leading to autoimmune responses and tissue damage. Modulating the expression of miRNAs involved in apoptotic pathways holds promising therapeutic potential for restoring proper apoptotic mechanisms and mitigating autoimmune responses in SLE. Future research should focus on elucidating the intricate regulatory networks involving miRNAs and their targets in apoptotic pathways, as well as developing miRNA‐based therapeutics for targeted modulation of apoptosis in immune cells implicated in SLE.

## Author Contributions

**Amin Azizan:** conceptualization, investigation, visualization, writing–original draft. **Elham Farhadi:** conceptualization, project administration, supervision, validation, writing–review and editing. **Majid Alikhani:** investigation, visualization; writing–review and editing.

## Conflicts of Interest

The authors declare no conflicts of interest.

## Data Availability

The authors have nothing to report.
